# The Doctrine of Animal Quinoidin

**Published:** 1869-01

**Authors:** 


### ARTICLE X.

### *The Doctrine of Animal Quinoidin*

A discussion on this subject in the Paris Biological Society, is referred to in the *Gazette Hebdomadaire*, from which we translate the following passages:

Two years ago Dr. Bence Jones, of the Royal Society of London, in the course of his researches on the time required for certain substances to traverse the tissues, was much embarrassed in regard to sulphate of quinia. To demonstrate the presence of this salt, Bence Jones and Doctor Dupré conceived the ingenious idea of subjecting the tissues to the action of acids, and examining the solutions thus obtained by means of the fluorescence produced by electric light—such fluorescence being presumed to indicate the presence of quinia. Great was their surprise to find the same fluorescence in animals that had not taken the quinia salts, as well as those that had. They came to the conclusion that there exists in the economy a substance capable of producing the same fluorescence as the sulphate of quinia. Bence Jones infers the existence of an animal quinoidin. He considers this new alkaloid an albuminoid derivative, which he places between casein and indigotin.

According to the same writer, this quinoidin plays an important part in the phenomena of nutrition: it acts as a conservative agent in retarding organic combustion. Having noticed the disappearance of the natural fluorescence in the urine of patients with intermittent fever, he regarded as possible the destruction of the quinoidin by malarious poison. Then, the more rapid combustion of the tissues would produce fever; and thus we may comprehend the mode of action of febrifuges such as quinia and arsenic, substances which, like the supposed quinoidin, have the property of retarding organic combustion.

Dr. Chalvet has confirmed the statements of Bence Jones, and demonstrated by his experiments that there exists in the tissues a substance capable of giving a fluorescence absolutely comparable to the phenomena of refrangibility produced by sulphate of quinia. He also demonstrated that this fluorescence often disappears in intermittent fevers; but he does not accept the interpretation of the English author, on the origin of the assumed quinoidin. He has, in fact, demonstrated that this fluorescent substance exists in most aliments, especially in wine and vegetable substances. He concludes, from his researches, that the assumed quinoidin is not an albuminoid derivative; that it is introduced into the organism with the ingesta; that it mingles with the humors and tissues like iron, but unlike iron, it is not generated in the organs. Possessing the property of rapid elimination by the secretions, we can comprehend how a diet somewhat reduced may cause its disappearance from the urine, and thus explain the asserted destruction of quinoidin by fever. Doctor Chalvet inclines to regard this substance as analogous to quinia, and as it is produced in infinitessimal quantity in almost all vegetables, its constant presence in the solids and fluids of all animals is easily explained. But this does not prove that the substance, though in minute quantity, is incapable of performing an important part in the phenomena of life; for we know that the mere presence of a few particles of certain matters, may develop by catalysis, forces of great relative magnitude.—*Pacific Medical and Surgical Journal.*

